# Clinical surveillance for human astrovirus in Monastir region, Tunisia

**DOI:** 10.1186/s12889-016-2726-5

**Published:** 2016-01-21

**Authors:** Abir Monastiri, Mahjoub Aouni, Susana Guix, Badereddine Mechri, Marc Lopez-Roig, Nabil Ben Salem Abid, Neji Gueddiche, Sabeur Hamami, Lamjed Boughzala, Jordi Serra-Cobo

**Affiliations:** 1 Laboratory of Contagious Diseases and Biologically Active Substances, LR99ES27, Faculty of Pharmacy, University of Monastir, Avicenne Street 5000, Monastir, Tunisia; 2 Department of Microbiology, Faculty of Biology, University of Barcelona, Av. Diagonal 643, 08028 Barcelona, Spain; 3 IRBIO and Department of Animal Biology, Faculty of Biology, University of Barcelona, Av. Diagonal 643, 08028 Barcelona, Spain; 4 Pediatric Department, University Hospital Fattouma Bourguiba, Monastir, Tunisia

**Keywords:** Human astrovirus, Clinical surveillance, RT-PCR, Cell culture, Tunisia

## Abstract

**Background/aims:**

Astroviruses (AstVs) are enteric viruses that can cause gastroenteritis in children. This study is part of monitoring the circulation of astroviruses in children hospitalized and/or outpatients for acute gastroenteritis at the primary care center of Ouerdanine or at the Pediatric Department of the University Hospital Fattouma-Bourguiba (Monastir, Tunisia). The aims of our study were to know the prevalence of human astrovirus in clinical samples of children, characterize the strains and evaluate the infectivity of isolated strains on cell culture.

**Methods:**

Fifty stool samples were collected from children under five years old in the region of Monastir (Tunisia) from October 2010 to June 2011. All specimens were subjected to RT-PCR amplification followed by sequencing and phylogenetic analysis.

**Results:**

The study shows a low prevalence of astrovirus (4 %) in children. The two positive samples obtained were HAstV type 3. Samples that were RT-PCR positive were cultured in CaCO-2 cells and the presence of infectious viral particles was confirmed. The phylogenetic analysis shows that the different HAstV-3 strains isolated in Tunisia are grouped into two clusters. The first cluster includes strains obtained in 2004, which belong to lineage HAstV-3a, while strains isolated in 2010 belong to lineage HAstV-3c.

**Conclusions:**

This study is part of monitoring the circulation of astroviruses in children younger than five years old from Monastir region, Tunisia. The results show low prevalence (4 %). All genotyped samples belonged to lineage HAstV-3c, which could be presently emerging. Two different lineages have been isolated in Tunisia: HAstV-3a in 2004 and HAstV-3c in 2010.

## Background

Astroviruses (AstVs) are enteric viruses that can cause gastroenteritis in children and a severe disease in immunocompromised and elderly people [1, 2]. The prevalence rate of human astrovirus (HAstV) infection ranged from 2 % to 9 % among children with diarrhea, although incidences over 60 % have also been reported [3]. The morbidity varies depending on the season, with higher infection during the winter in temperate climates and the rainy season in tropical regions [4]. The main symptom of astrovirus infection is watery diarrhea, which is often associated with vomiting, fever, and abdominal pain [2].

Astroviruses are a non-enveloped positive-strand RNA viruses belonging to the *Astroviridae* family [5]. HAstVs are genetically and antigenetically heterogeneous and include three major groups [3]. The first includes the classic eight serotypes of HAstVs (HAstV-1 to HAstV-8). The other two groups (MLB and VA clades), identified in 2009, are also prevalent but their pathogenic role in humans requires further characterization [3]. Epidemiological surveys on classic HAstVs show that HAstV-1 is the most common serotype identified worldwide in children [6, 7], while HAstV-6, and −7 are less frequent [8, 9]. A precedent study, performed in Tunisia, showed the predominance of genotype HAstV-1 [10].

The aims of our study were to know the prevalence of HAstVs in clinical samples of children from Tunisia obtained in 2010–2011, characterize the strains and evaluate the infectivity of isolated strains.

## Methods

### Clinical samples

Fifty stool samples were collected from children younger than five years old hospitalized and/or seeking medical care for acute gastroenteritis at the Primary Care Center of Ouerdanine or at the Pediatric Department of the University Hospital Fattouma-Bourguiba (Monastir, Tunisia). Samples were obtained from October 2010 to June 2011 and within 24 h following hospitalization or visit. The main clinical symptoms of the patients were: diarrhea, fever (>37 °C) and vomiting. All samples were free from bacterial and parasitic enteric pathogens (tested by routine hospital procedure).

The study protocol was approved by the Ethics and Research Committee of University Hospital Fattouma-Bourguiba (Monastir, Tunisia) and written informed consent was obtained from the parents of the 50 study participants.

### Virus concentration

Stools were suspended (10 %, w/v) in phosphate-buffered saline (PBS) containing 2 M NaNO_3_, 1 % bovine serum albumin; fraction V, and 0.1 % Triton X- 100 (pH 7.2). After vortexing samples were centrifuged for 15 min at 12,000 rpm and the supernatant was stored at −70 °C.

### Detection of Astrovirus RNA

RNA was extracted from 400 μl of stool suspension using phenol/chloroform and ethanol elution method. RNA was eluted in 30 μl of DEPC-treated water (Invitrogen). RT-PCR was carried out using primers Mon269 and Mon270 [11] targeting a 449 bp region of the capsid protein gene (ORF2), whereas primers AV1 and AV2 were used to amplify a 90 bp highly conserved region at the 3′ end of astrovirus genome [12]. The reverse transcription step was performed by using 5 μl of RNA added to 10 μM of primer Mon270, 1 mM of each dNTP, 2 mM DTT and 1.25 U of M-MLV reverse transcriptase (Invitrogen, Tunisia) in a 25 μl total reaction volume containing 25 mM Tris–HCl (pH 8.3), 20 mM KCl and 3 mM MgCl_2_. The cDNA was synthesised at 42 °C for 60 min. For the PCR reaction, 5 μl of the RT product was added to 20 μl reaction mixture containing 2 mM MgCl_2_, 10 mM of each dNTP, 10 μM of each primer Mon269 and Mon270 and 1.25 U of Taq polymerase (Biomatik, Tunisia). The 25 μl reaction mixture was amplified using the following cycling conditions: 40 cycles of amplification at 94 °C for 1 min, at 50 °C for 1 min and at 72 °C for 1 min. For the second RT-PCR using primers set AV1/AV2, the reaction mixture was the same as the first reaction but with the following amplification conditions: 30 cycles at 90 °C for 1 min, at 40 °C for 2 min and at 72 °C for 1 min. A total of 10 μl of RT-PCR product was analyzed on a 2 % agarose gel. Gel images were recorded with the Gel Doc 2000 system (Bio-Rad Laboratories). PCR products were purified using ExoASP-IT® (Affymetrix) and sequenced using the ABI BigDye® Terminator v3.1 Cycle Sequencing Kit (Applied Biosystems) and ABI 3730xl DNA Analyzer (Applied Biosystems).

### Phylogenetic analysis

The sequences obtained were blasted against Genbank using Sequencher 10.1 software. A phylogenetic tree was constructed with Kimura-two parameter model [13] and neighbor-joining methods (5,000 bootstrap replications) using Mega 6 software [14].

### Cell culture and inoculation

CaCO-2 cell were grown in DMEM-F12 medium supplemented with 2 mM glutamine, 10 % fetal bovine serum (FBS), and 1 % antibiotic mixture (100 units Penicillin ml^−1^/100 mg Streptomycin ml^−1^). For inoculation, stool suspensions (10 %, v/v) were prepared in DMEM-F12 supplemented with 1 % antibiotic mixture. Suspensions were kept at 4 °C for 24 h, centrifuged for 30 min at 7,000 rpm and filtered (0.22 μm filter). Prior to inoculation, cells were washed with serum-free DMEM-F12 and infected with the virus in the presence of 10 μg/ml of trypsin. After 1 h, inoculums was replaced with fresh DMEM-F12 containing 2 % FBS. RNA extraction was performed from 200 μl of culture supernatant harvested at different times post-infection.

## Results

### Astrovirus detection and typing

**Fig. 1:**
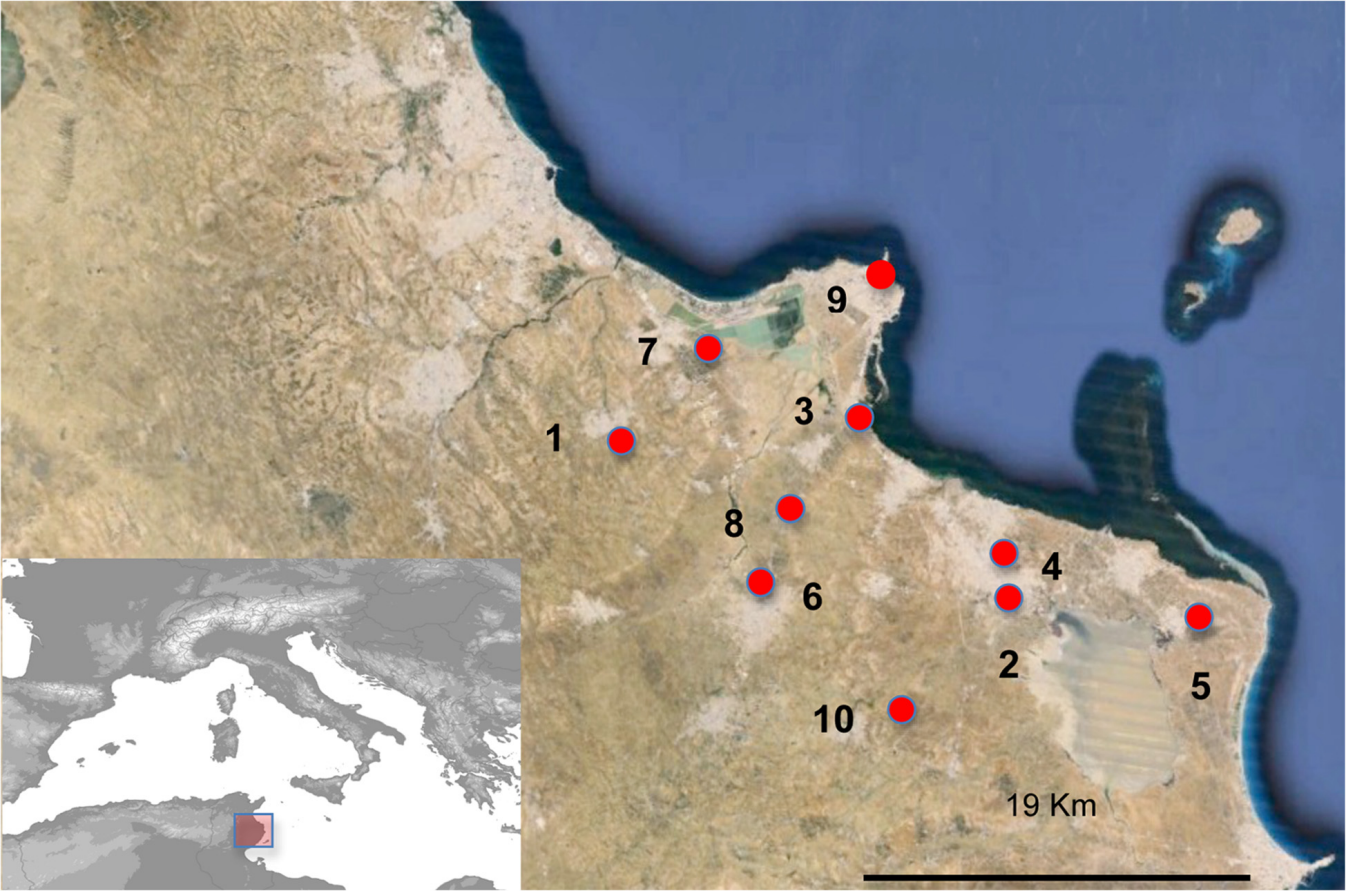
Geographic distribution of infantile gastroenteritis cases in the region of Monastir (Tunisia). 1: Ouerdanine; 2: Moknine; 3: Khniss; 4: Ksar Hellal; 5: Bekalta; 6: Jemmal; 7: Sahline; 8: Menzel Ennour; 9: Monastir; 10: Beni Hassen

**Fig. 2:**
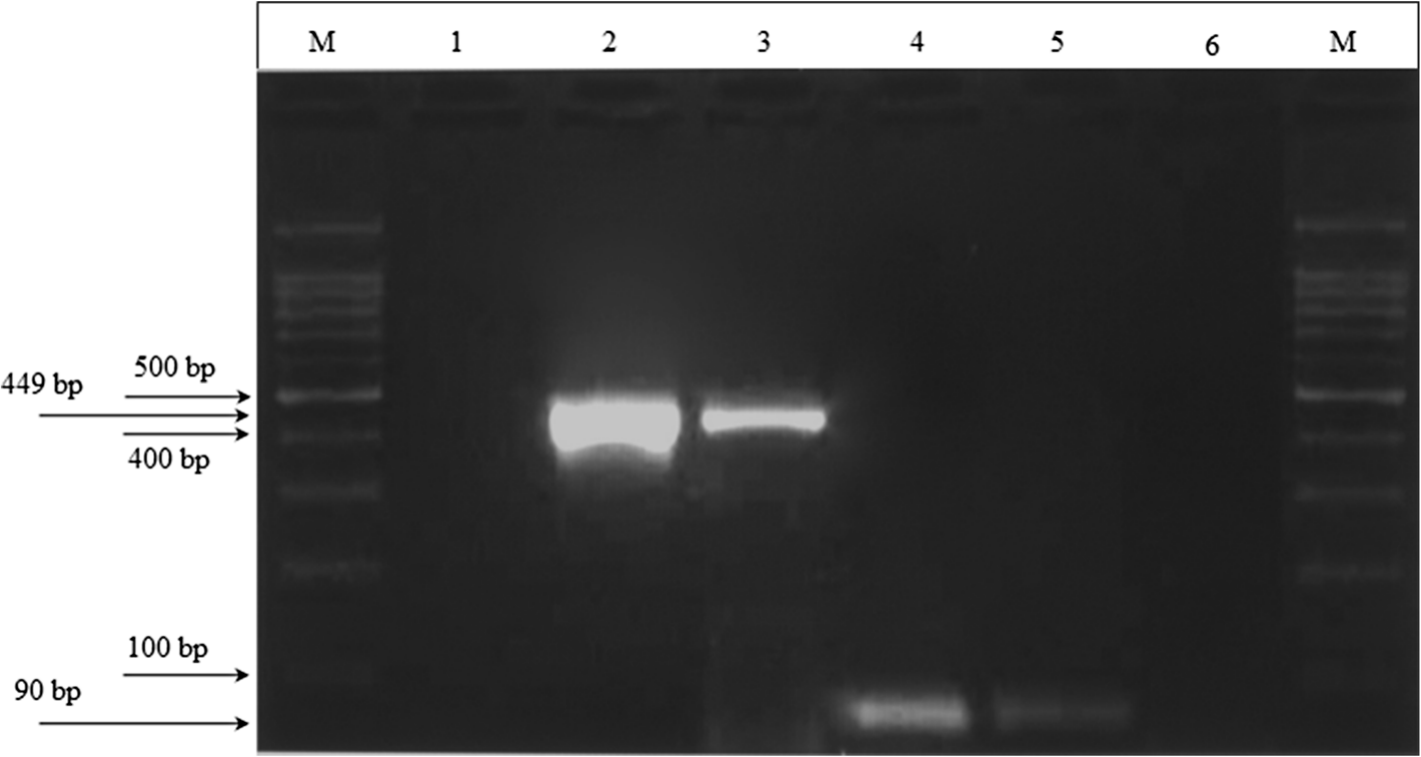
Detection of astrovirus RNA in two positive stool samples. M: DNA marker 100 bp; Lane 1: Mon primers negative control (DEPC-treated water); Lane 2 and 3: PCR results of stool samples Tunisia 6 and Tunisia 7 using Mon269/Mon270 primers; Lane 4 and 5: PCR results of stool samples Tunisia 6 and Tunisia 7 using AV1/AV2 primers; Lane 6: AV primers negative control (DEPC-treated water); M: DNA marker 100 bp

We analyzed samples from 19 girls and 31 boys aged to 2 and 60 months (average 14.16 ± 13.69 months) that came from 10 different localities (Fig. 1). Twenty-eight children were outpatients and 22 inpatients. Two samples (4 %), obtained in October 2010 from two inpatient boys aged 3 and 6 months that came from Ksar Hellal and Khniss localities, respectively, were positive (Fig. 2). The boy aged 3 months showed only diarrhea and the first symptoms appeared two days before the visit to the doctor. The patient aged 6 months showed diarrhea associated with fever (39 °C), vomiting and the symptoms appeared one day before the visit to the hospital.

**Fig. 3:**
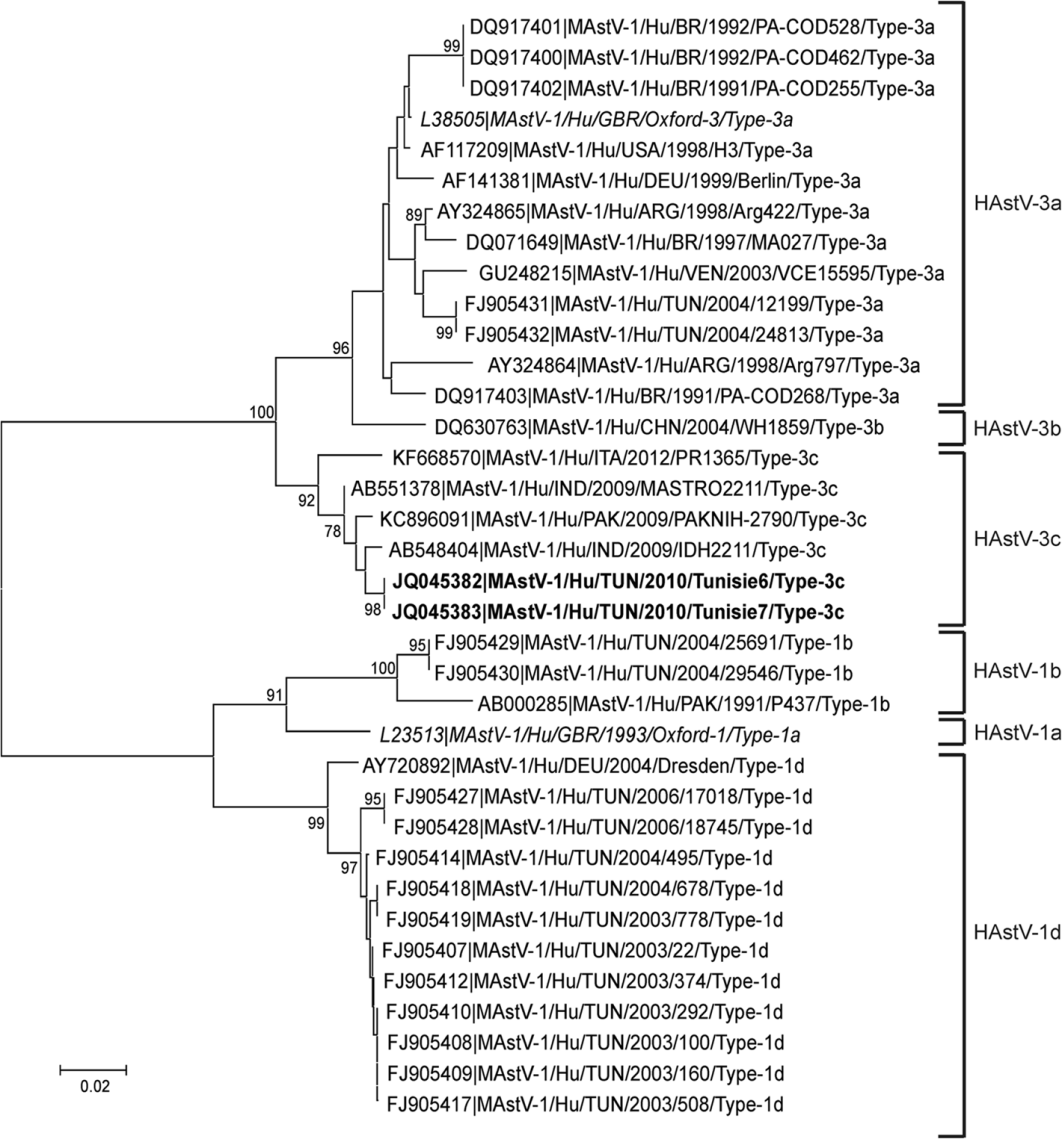
NJ phylogenetic tree obtained from sequences

The two sequences obtained from ORF2 belonged to lineage HAstV-3c [15]. They shared 98.6 % and 97.9 % of nucleotide identity with the HAstV-3c reference strains KC896091 and AB551378 isolated in Pakistan and India in 2009, respectively. Our sequences shared 92.2 % of homology with the sequences of HAstV-3 obtained in 2004 at Tunisia (FJ905431, FJ905432S) [16] (Fig. 3). Sequences obtained were submitted under accession numbers: [GenBank: JQ045382, GenBank: JQ045383].

### Astrovirus passage in CaCO-2 cells combined to RT-PCR

The two positive samples by RT-PCR were cultured in CaCO-2 cells and the presence of infectious viral particles was confirmed. RNA extracted from culture supernatant harvested at different times post-infection was amplified by RT-PCR using primers Mon269/Mon270. RT-PCR band intensity increased over time, which could suggest virus propagation in the infected monolayers.

## Discussion

We report the results of a clinical surveillance in children hospitalized and/or outpatients for acute gastroenteritis in the Monastir region (Tunisia). This study was performed three years and half after the last clinical surveillance in the region [16]. The low prevalence of astrovirus obtained in our study (4 %) is consistent with the study conducted in the Monastir region from January 2003 to April 2007 (3.6 %) [17], as well as in other countries such as in France (6 %), Italy (3.1 %), Spain (4.9 %) and India (5.8 %) [3]. The results confirm once again the low incidence of astrovirus in gastroenteritis of children under 5 years old, in concordance with precedent studies made in Tunisia and Spain [3, 16].

HAstV-1 is the most common serotype identified in children in most countries where the astrovirus was analyzed [7, 8,18-19]. Previous Tunisian study also showed a predominance of the HAstV-1 (85.6 %) over HAstV-3 (14.3 %) [10]. All the HAstVs obtained in 2003 were of serotype 1, while in 2004 the prevalence of HAstV-3 was relatively high (36 %). In 2005, all HAstVs found in Tunisia were HAstV-1 [10]. However, the two positive samples obtained in our study were HAstV-3. Different authors suggest a lack of heterotypic immunity between the different antigenic types of HAstV, which could be responsible for the changes in serotype distribution observed in consecutive years [18, 20].

The phylogenetic analysis shows the occurrence of two different lineages of HAstV-3 in Tunisia. Strains obtained in 2004 [10] belong to lineage HAstV-3a, whilst our 2010 strains belong to lineage HAstV-3c. The Tunisian strains of the two lineages differ in 7.8 % of nucleotides. Interestingly, many of the HAstV-3 recently reported are of the 3c lineage [15, 21, 22], suggesting that this lineage may be presently emerging.

PCR techniques are unable to differentiate between viral RNA associated to infectious or non-infectious particles. As shown in other studies, CaCO-2 cell culture combined with RT-PCR constitutes a reliable tool for evaluation of HAstV infectivity and increases PCR sensitivity as well [23, 24].

## Conclusions

This study is part of a survey on the circulation of astroviruses in children  hospitalized and/or outpatients for acute gastroenteritis in the Monastir region, Tunisia. The results show a low prevalence of astrovirus (4 %) in children younger than five years old. All genotyped samples belonged to lineage HAstV-3c. The results are in concordance with a lack of heterotypic immunity between the different antigenic types of HAstV. The phylogenetic analysis shows that Tunisian strains isolated in 2004 and 2010 belong to two different lineages and suggest that lineage HAstV-3c may have emerged and replaced HAstV-3a strains over time.
